# Effect of bacterial inoculation on co-composting of lavender (*Lavandula angustifolia* Mill.) waste and cattle manure

**DOI:** 10.1007/s13205-021-02860-2

**Published:** 2021-05-31

**Authors:** Babett Greff, Jenő Szigeti, Ágnes Varga, Erika Lakatos, András Sáhó, László Varga

**Affiliations:** 1 Department of Food Science, Faculty of Agricultural and Food Sciences, Széchenyi István University, 15-17 Lucsony Street, Mosonmagyaróvár, 9200 Hungary; 2 Kisalföldi Agricultural Ltd, Fő út 1., Nagyszentjános, 9072 Hungary

**Keywords:** Compost, Lavender, Herb, *Cellulomonas*, *Streptomyces*

## Abstract

The primary purpose of this study was to investigate the influence of *Cellulomonas flavigena* and *Streptomyces viridosporus*, as a bacterial inoculant, on the compostability of post-extraction lavender waste. The major physicochemical, microbiological, and biological properties of the composting materials were monitored for 161 days. The technology developed was shown to improve the compostability of recalcitrant herbal residues. The use of lavender waste beneficially affected the composting process by extending the thermophilic phase, accelerating the degradation of organic matter, and elevating the viable counts of useful microorganisms; however, adverse effects were also observed, including an increased carbon-to-nitrogen ratio (19.05) and a decreased germination index (93.4%). Bacterial inoculation was found to preserve the nitrogen content (2.50%) and improve the efficiency of biodegradation. The *Salmonella*- and *Escherichia coli*-free final composting products were mature, stable, and ready for soil application. To the authors’ knowledge, no previous research has investigated the compostability of lavender waste. Likewise, this is the first study that has used strains of *C. flavigena* and *S. viridosporus* in combination to facilitate a composting process.

## Introduction

Herbs are extensively used for their medicinal, antipathogenic, aromatic, and culinary properties by the pharmaceutical, cosmetic, and food industries (Greff et al. 2021; Sánchez-Vioque et al. 2013; Semeniuc et al. 2017). Their beneficial effects have primarily been attributed to their essential oil (EO) content. These volatile oils are complex mixtures of diverse components including terpenes, phenolic compounds, ketones, ethers, esters, aldehydes, and alcohols obtained from various herbal parts such as seeds, roots, rhizomes, leaves, fruits, flowers, buds, and barks (Sánchez-Vioque et al. 2013; Shaaban et al. 2012).

Lavender (*Lavandula angustifolia* Mill.), an evergreen bushy shrub, is one of the major species of the family *Lamiaceae*. It is commercially cultivated in many countries, e.g., France, Hungary, Bulgaria, China, the UK, the USA, Australia, Portugal, and Spain (Rashed et al. 2019; Yazdani et al. 2013). Lavender EO is among the best-selling over-the-counter herbal remedies for stress and anxiety with a global yearly production of approximately 200 tonnes (Lesage-Meessen et al. 2018; López et al. 2017). The rapidly growing EO production, the low EO content of herbs, and the inefficiency of extraction processes result in huge amounts of herbal residues rich in biologically active compounds worldwide (Greff et al. 2021; Ratiarisoa et al. 2016; Saha and Basak 2020; Slavov et al. 2020). In France alone, over 20,000 tonnes of solid residues of lavandin- and lavender-distilled straws are generated annually (Lesage-Meessen et al. 2018).

The traditional treatments (i.e., incineration or disposal in landfills) of herbal residues are environmentally unfriendly and expensive (de Elguea-Culebras et al. 2016; Deka et al. 2011; Ibrahim et al. 2017; Tian et al. 2017; Yohalem and Passey 2011). In addition, they are a waste of precious resources (Tian et al. 2017). In recent years, recycling and resource recovery have emerged as the most promising options for sustainable management of herbal wastes (Singh and Suthar 2012). Composting is an effective way to stabilize and reuse post-extraction wastes through microbial decomposition of biodegradable materials under controlled conditions (Zhang et al. 2011). However, the post-extraction residues of lavender can be difficult to biotransform because they are rich in cellulose, hemicellulose, lignins, and other components including lactones, terpenes, and phenolic compounds (Lesage-Meessen et al. 2015). Various biological methods, such as the addition of co-substrates (co-composting) or microbial inoculation during composting, may be used to overcome these difficulties. Co-composting with herbal wastes tends to increase the rate of organic matter (OM) degradation by microorganisms and it often results in improved compost quality with high antipathogenic properties (Zhou et al. 2016, 2018). However, the use of herbal residues may at times negatively influence the composting process because an increased lignocellulose content and the remaining secondary metabolites can inhibit nutrient cycling, litter decomposition, and seed germination (Bohacz 2019a; De Martino et al. 2010; Guénon et al. 2017; Shang et al. 2016; Zhang et al. 2019). Inoculation of the waste material with specific microbial cultures may stimulate the biological degradation of OM, thus improving final compost quality (Huang et al. 2015).

Since only limited information is available in the literature on the compostability of herbal residues, the objective of this study was to produce a mature compost by utilizing the distillation waste of lavender, cattle manure, and straw. A mixed culture of *Cellulomonas flavigena* and *Streptomyces viridosporus* strains was applied to improve the co-composting process and the quality of the end product. Temperature, pH value, moisture content, and microbial density (i.e., viable counts of mesophilic and thermophilic microorganisms, cellulose-degrading bacteria, streptomycetes, and fungi) were monitored in the compost bins over a period of 161 days. In addition, OM, total organic carbon (TOC), total nitrogen (TN), and acid-insoluble lignin contents, carbon-to-nitrogen (C/N) ratio, biodegradability coefficient, germination index (GI), and the presence or absence of potentially pathogenic enterobacteria (*Escherichia coli* and *Salmonella* spp.) were also determined in the final products to evaluate compost maturity. To our knowledge, no previous research has investigated the compostability of post-extraction lavender waste. Likewise, as far as we know, this is the first study that has used strains of *C. flavigena* and *S. viridosporus* in combination to facilitate a composting process.

## Materials and methods

### Preparation of composts and sampling

Outdoor pilot-scale composting trials were carried out at Kisalföldi Agricultural Ltd (Nagyszentjános, Hungary) from September 2019 through February 2020, for a total of 161 days. Compost bins (1 m^3^) were made from pallets and were covered with mesh and insulated with styrofoam. Each bin contained 250 kg of waste material as follows:

- Control compost (CC): 90% cattle manure and 10% barley straw.
- Control lavender waste compost (CLC): 60% lavender waste, 30% cattle manure, and 10% barley straw.
- Lavender waste compost with bacterial inoculation (LCI): 60% lavender waste, 30% cattle manure, and 10% barley straw inoculated with the mixed culture of *C. flavigena* NCAIM B.01383 and *S. viridosporus* NCAIM B.02369 (for more details, see subsection entitled* Preparation of lavender waste compost with bacterial inoculation*).

The composition of raw materials used to make the composts is shown in Table 1. Post-extraction lavender waste, cattle manure, and barley straw batches were provided by the same supplier (Kisalföldi Agricultural Ltd, Nagyszentjános, Hungary). It should also be noted that lavender was harvested from the same location and the lavender biomass was produced by water distillation.

**Table 1:** Major parameters (on dry weight basis) of raw materials used for the composting trials

Parameter	Post-extraction lavender waste	Cattle manure	Barley straw
Moisture (%)	52.84 ± 5.27	73.43 ± 1.14	25.62 ± 7.54
Total solids (%)	47.16 ± 5.27	26.57 ± 1.14	74.38 ± 7.54
Organic matter (%)	91.91 ± 0.69	84.12 ± 0.43	93.52 ± 0.11
Total organic carbon (%)	51.06 ± 0.38	46.73 ± 0.24	51.96 ± 0.06
Total nitrogen (%)	1.37 ± 0.04	1.68 ± 0.08	0.55 ± 0.01

All values are means ± SD based on three observations

Each mixture was carefully homogenized, and the initial moisture content was adjusted to 55% by water addition. Over the months, the content of the compost bins was turned over manually three times to aerate the mixture. Lost water was replaced by adding tap water to the bins.

On days 0, 8, 15, 21, 42, 56, 78, and 161, subsamples were taken from five representative points in the bins. These subsamples were mixed thoroughly (Ryckeboer et al., 2003) to obtain homogeneous samples of approximately 500 g each, which were then divided into two parts. One part was used for chemical analysis after natural air drying, whereas the other part of the fresh sample was used immediately for determination of microbiological properties, pH value, and GI.

#### Preparation of lavender waste compost with bacterial inoculation

The mixed waste consisted of solid residues of extracted lavender (60%) and cattle manure (30%). In addition, barley straw (10%) was used as a bulking agent to give sufficient air-filled porosity and structure to the composting piles and maintain the required aerobic conditions (Adhikari et al. 2009). Cellulolytic and lignin-degrading strains of *C. flavigena* (NCAIM B.01383) and *S. viridosporus* (NCAIM B.02369), respectively, were obtained from the National Collection of Agricultural and Industrial Microorganisms (NCAIM; Budapest, Hungary) and were used as a bacterial inoculant. Before inoculation, the bacterial pure cultures were cultivated separately, at 30 °C for 100 h, in Dubos Salts Broth supplemented with carboxymethyl cellulose (CMC) as the sole carbon source. This liquid culture medium contained (per liter) 0.5 g of NaNO_3_, 0.5 g of K_2_HPO_4_, 0.5 g of MgSO_4_ × 7 H_2_O, 1.0 g of KCl, 0.01 g of FeSO_4_ × 7 H_2_O, 2.0 g of yeast extract, and 10.0 g of CMC. The incubated suspensions of *C. flavigena* NCAIM B.01383 (7.1 × 10^8^ CFU/mL) and *S. viridosporus* NCAIM B.02369 (6.0 × 10^7^ CFU/mL) were mixed at a ratio of 1:1. On day 8 of the composting process, 1 part of inoculant was diluted with 20 parts of water, which was then sprinkled on the composting material at a concentration of 8% (v/w) while the contents of the bins were turned. The even distribution of the bacterial amendment was thus ensured.

### Physicochemical analysis

Temperature was measured daily with a stainless-steel compost thermometer (Electronic Temperature Instruments, Worthing, UK) inserted in the center of the compost bins. pH value was determined in a 1:10 (w/v) water-soluble extract using a Jenway 3510 pH-meter and combined glass electrode (Keison Products, Chelmsford, UK) standardized with pH 7.00 and 4.00 standard buffer solutions (Merck, Darmstadt, Germany). Moisture content (%) was assessed by oven-drying the samples at 105 °C to constant weight, and OM content (%) was determined by gravimetric loss on ignition (Yeoh et al. 2011). From OM results, TOC percentages and biodegradability coefficients (*K*_b_) were calculated as follows (Kebibeche et al. 2019; Rashad et al. 2010):

1 $${\text{TOC}}\;\left( \% \right)\; = \;\frac{{{\text{OM}}\left( \% \right)}}{1.8},$$

2 $$K_{{\text{b}}} = \;\frac{{\left( {{\text{OM}}_{{\text{b}}} - \;{\text{OM}}_{{\text{e}}} } \right)\; \times \;100}}{{{\text{OM}}_{{\text{b}}} \; \times \;\left( {100\; - \;{\text{OM}}_{{\text{e}}} } \right)}},$$

where OM_b_ and OM_e_ are organic matter contents at the beginning and end, respectively, of the composting process. TN content (%) was determined by the combustion method with a Rapid N Cube analyzer (Elementar Analysensysteme, Langenselbold, Germany) (Su et al. 2016). C/N ratio was calculated based on TOC and TN values. Acid-insoluble lignin, a.k.a. Klason lignin, content was determined according to TAPPI standard method T 222 (TAPPI T 222 om-02 2002) and the final residues were corrected for ash.

### Microbiological analysis

For enumeration of microorganisms, each compost sample was diluted by mixing 10 g (wet weight) with 90 mL of sterile saline solution (0.85% NaCl) and homogenized thoroughly in a Stomacher 400 Circulator (Seward, Worthing, UK). Further decimal dilutions were made up to 10^–9^. The pour plate method was used to enumerate microbes, except for *Salmonella* spp. where the qualitative presence–absence test was performed, under aerobic conditions. Colony-forming units (CFU), expressed as log_10_ per gram, were used to report changes in viable counts of various microbial groups in different types of composts.

Plate Count Agar (Biolab, Budapest, Hungary) was used to enumerate mesophilic and thermophilic microorganisms. The inoculated plates were incubated at 30 °C (mesophiles) or 55 °C (thermophiles) for 72 h.

Mesophilic and thermophilic cellulose-degrading bacteria were enumerated in Dubos Salts Agar supplemented with CMC (Rajoka and Malik 1997). The inoculated agar plates were incubated for 72–120 h at 30 °C and 55 °C, respectively. Cellulase activity was detected by flooding the CMC-containing plates with 0.1% (w/v) Congo Red solution (Merck) for 30 min and then rinsed with 1 N NaCl.

The viable counts of mesophilic and thermophilic streptomycetes were determined in Gauze’s Synthetic Medium No. 1 following 72–120 h of incubation at 30 °C and 55 °C, respectively. The solid culture medium contained (per liter) 20.0 g of soluble starch, 1.0 g of KNO_3_, 0.5 g of NaCl, 0.5 g of MgSO_4_ × 7 H_2_O, 0.5 g of K_2_HPO_4_, 0.01 g of FeSO_4_ × 7 H_2_O, and 15.0 g of agar. Since this medium is not strictly selective for streptomycetes, only colonies with aerial mycelia were counted (Ryckeboer et al. 2003).

Rose Bengal Chloramphenicol Agar (Biolab) was used for enumeration of mesophilic and thermophilic fungi. Incubation was carried out at 25 °C and 55 °C, respectively, for 72–120 h.

*Escherichia coli* was enumerated in ChromoBio Coliform Agar (Biolab) following 24–48 h of incubation at 37 °C.

For detection of *Salmonella* spp., compost samples each weighing 25 g were homogenized with 225 mL of Buffered Peptone Water (Biolab) and incubated at 37 °C for 18 h. From this pre-enrichment medium, 0.1 mL was transferred to 10 mL of Rappaport–Vassiliadis Broth (Biolab) and further incubated at 41.5 °C for 24 h. A loopful of selectively enriched sample was streaked onto Xylose Lysine Deoxycholate Agar (Biolab) and incubated at 37 °C for 24 h. Red and pink colonies with or without a black center were considered presumptive *Salmonella* spp., which were then subcultured and their identity was confirmed by appropriate serological and biochemical tests (ISO 6579-1 2017).

### Phytotoxicity test

A seed germination assay was conducted under laboratory conditions to confirm the maturity of composts. Aqueous extracts of compost samples were prepared by shaking 10 g of sample in 100 mL of distilled water for 1 h at 25 °C and 200 rpm, using a water bath shaker (Gyrotory Model G76D; New Brunswick Scientific, Edison, NJ, USA), and the mixtures were then filtered. Seeds of *Brassica rapa* subsp. *chinensis* were used for the experiments carried out in triplicate. Ten seeds each were placed on sterile filter paper discs (Merck) in 90 mm diameter plastic Petri dishes (Greiner Bio-One, Mosonmagyaróvár, Hungary). Filter papers were previously moistened with 5 mL aliquots of each compost extract. Distilled water served as a control. After incubation at 25 °C for 72 h in the dark, the number of germinated seeds was determined. GI was calculated as described by Zucconi et al. (1981).

### Statistical analysis

Most of the results presented in this paper are the means ± SD of three replicate analyses. The data were subjected to one-way analysis of variance (ANOVA). Significance of difference was set at *p* < 0.05 in all cases.

## Results and discussion

### Changes in physicochemical properties during composting

**Fig. 1:**
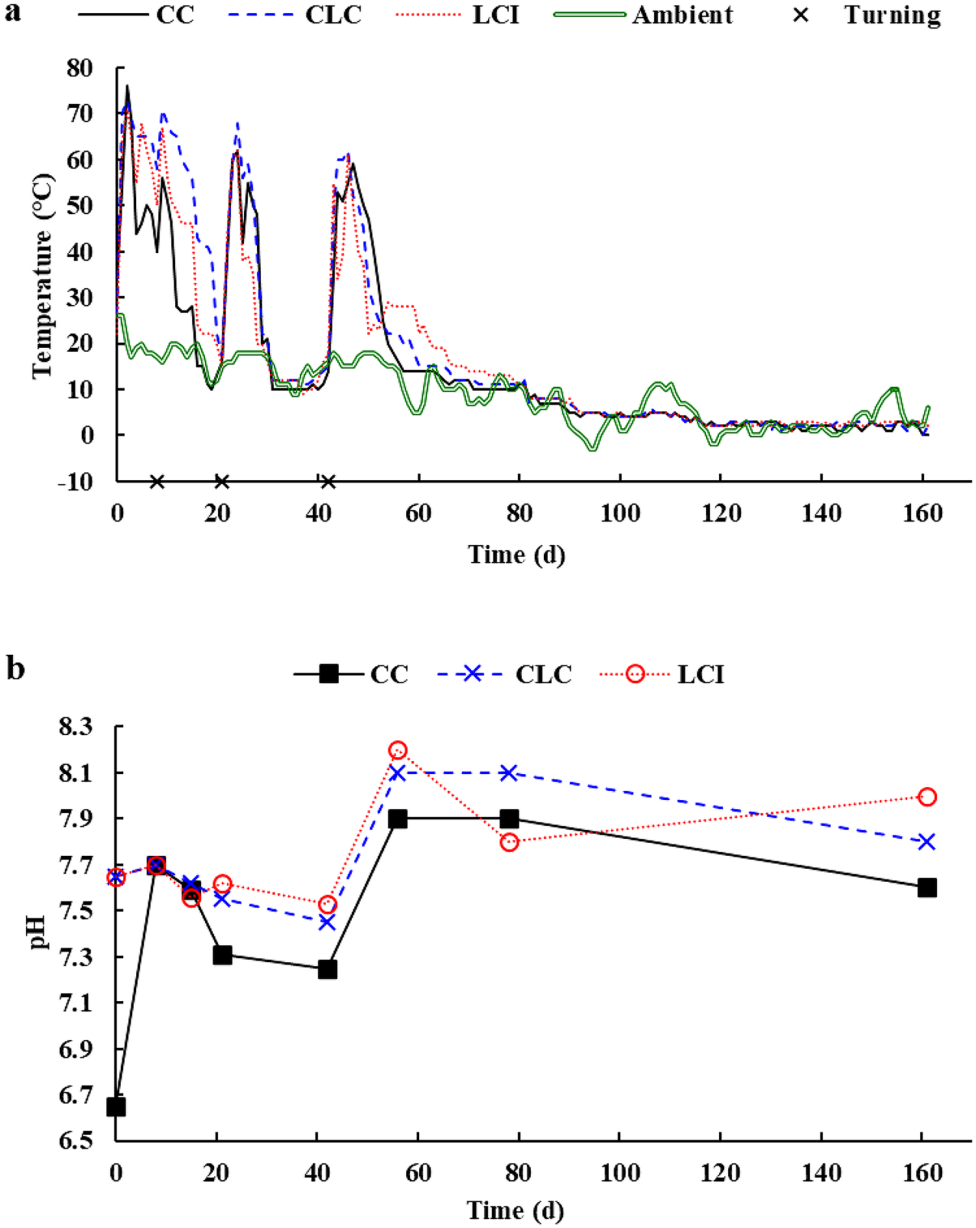
Changes in core temperature (**a**) and pH value (**b**) during the composting process (*CC* control compost, *CLC* control lavender waste compost, *LCI* lavender waste compost with bacterial inoculation)

Changes in temperature of the three types of composts are shown in Fig. 1a. Once the composting process started, core temperatures rose steeply, reaching the thermophilic phase (> 50 °C) within a day in all compost bins. Temperature remained over 60 °C for 3, 6, and 7 days in CC, LCI, and CLC piles, respectively, and then a fast decline was observed. However, when the composting material was turned over, thermal reactivation occurred. All things considered, the thermophilic phase lasted for 15, 25, and 16 days in CC, CLC, and LCI, respectively. The temperature of the control pile containing no lavender waste (CC) reached its peak of 76 °C on day 3, whereas the corresponding values for CLC and LCI were 73 °C and 71 °C, respectively, at the same stage. The final cooling phase started on day 50 and, as a result, the core temperature of all three types of composts gradually decreased to 0–2 °C. Although the bins were insulated, the temperature of composts started to decline rapidly due to low ambient winter temperatures.

It should be noted that changes in compost temperature generally reflect the activity of microorganisms and the overall progress of composting (Li et al. 2018). The thermophilic phase is a crucial part of the composting process because cellulose and various phytotoxic compounds are degraded by thermophilic bacteria (Zhong et al. 2018). Furthermore, temperatures must exceed 55 °C for at least three consecutive days to ensure proper disinfection (Zeng et al. 2010; Zhong et al. 2018). The results showed that CC failed to comply with this requirement. In contrast, the use of lavender waste for composting was shown to extend the thermophilic phase, which was, however, 9 days shorter in LCI than in CLC, indicating that inoculation with *C. flavigena* and *S. viridosporus* could not promote the composting process in this regard.

Microbial activity largely depends on pH conditions in the composting environment. Most microorganisms, especially bacteria, grow best around neutral pH values (Pandey et al. 2009). The pH of the three types of composts ranged between 6.65 and 8.20 over the entire composting period (Fig. 1b). The presence of lavender waste in the compost increased the initial pH value of CLC and LCI, but the pH of CC also rose rapidly from 6.65 to 7.70 by the end of the first week. This must have been due to the production of ammonia from decomposition of proteins and amino acids. With the progress of composting, pH gradually decreased in all three treatments because of CO_2_ emissions from degradation of OM (Jiang et al. 2015), nitrification, and formation of low molecular weight fatty acids (Gou et al. 2017). As from day 21, pH followed the same trend for CC and CLC. After turning over on day 42, a considerable increase in pH of all three types of composts was observed, which could be explained by additional protein mineralization and decomposition of organic acids (Voběrková et al. 2017). Between days 56 and 78, the bacterial additive in LCI increased the decomposition rate of organic compounds and the production organic acids. At completion of composting, the final pH values were in the range of 7.6–8.0, thus complying with the recommended level of < 9 (Gou et al. 2017).

Changes in OM, TOC, and TN contents and C/N ratio during the composting process were affected by the initial composition of composts (Table 2). With the progress of time, OM content declined (*p* < 0.05) in all composting bins. The initial OM percentages ranged from 86.73 to 87.27 (on dry weight basis), whereas the final values were between 76.19 and 80.90%. TOC content showed a similar downward trend with advancement of composting time. Final OM and TOC percentages were significantly lower (*p* < 0.05) in LCI than in CC and CLC. As a result, LCI had the highest *K*_b_ value (0.53), followed by CLC (0.46) and CC (0.35). These results clearly indicate that the combined use of lavender waste and bacterial inoculant could enhance the depletion of OM in compost.

**Table 2:** Major chemical properties (on dry weight basis) of different types of composts at the beginning and end of the composting process

Parameter	Day	CC	CLC	LCI
Organic matter (%)^d^	Initial	86.73 ± 0.99^a^	87.27 ± 0.14^a^	87.27 ± 0.14^a^
	Final	80.90 ± 0.46^a^	78.74 ± 0.19^b^	76.19 ± 0.23^c^
Total organic carbon (%)^d^	Initial	48.18 ± 0.55^a^	48.48 ± 0.08^a^	48.48 ± 0.08^a^
	Final	44.94 ± 0.26^a^	43.75 ± 0.10^b^	42.33 ± 0.13^c^
Biodegradability coefficient (*K*_b_)		0.35	0.46	0.53
Total nitrogen (%)^d^	Initial	1.49 ± 0.13^a^	1.47 ± 0.02^a^	1.47 ± 0.02^a^
	Final	2.55 ± 0.02^a^	2.30 ± 0.01^b^	2.50 ± 0.03^a^
Carbon-to-nitrogen ratio^d^	Initial	32.43 ± 2.45^a^	32.99 ± 0.34^a^	32.99 ± 0.34^a^
	Final	17.60 ± 0.15^b^	19.05 ± 0.06^a^	16.91 ± 0.18^c^
Acid-insoluble lignin (%)^d^	Initial	15.05 ± 0.58^a^	15.60 ± 0.74^a^	15.60 ± 0.74^a^
	Final	14.09 ± 0.33^ab^	14.16 ± 0.68^a^	12.66 ± 0.99^b^

*CC* control compost, *CLC* control lavender waste compost, *LCI* lavender waste compost with bacterial inoculation

^a–c^Means within a row without a common lowercase superscript differ (*p* < 0.05)

^d^Values are means ± SD based on three observations

As far as TN content is concerned, the final percentages were higher (*p* < 0.05) than the initial ones, which must have been due the above-mentioned carbon losses (Gou et al. 2017) and the concentrating effect of weight loss associated with OM mineralization (Kausar et al. 2013). The composts were found to differ (*p* < 0.05) in final TN content because the use of lavender waste decreased the concentration of nitrogen in CLC (2.30%) compared to CC (2.55%). However, a higher final TN content (*p* < 0.05) was observed in LCI than in CLC due to the concentrating effect of organic matter mineralization (Sánchez-Mondero et al. 2001).

C/N ratio is a commonly used parameter for the evaluation of compost maturity (Jusoh et al. 2013). The initial values were adjusted to between 30 and 35 (i.e., 32.43 for CC and 32.99 for CLC and LCI) with cattle manure and barley straw. After 161 days of composting, initial C/N ratios decreased by 45.7%, 42.3%, and 48.7% in CC, CLC, and LCI, respectively. The lowest final value of 16.91 (*p* < 0.05) belonged to LCI, which supports the notion that ligninolytic and cellulolytic microorganisms can accelerate the composting process. Similar results were reported by other researchers (Abdel-Rahman et al. 2016; Li et al. 2020; Liu et al. 2011; Selvamani et al. 2019; Wan et al. 2020; Zhao et al. 2017). Lignin biodegradation is also a crucial process determining the humification and stabilization of the final products and, ultimately, the length of time needed for the completion of composting (Gou et al. 2017). The initial acid-insoluble lignin content of composts was reduced by 6.4–18.8% until the end of the composting process. The lowest final value of 12.66% was measured in LCI.

### Changes in microbial viability during composting

**Fig. 2:**
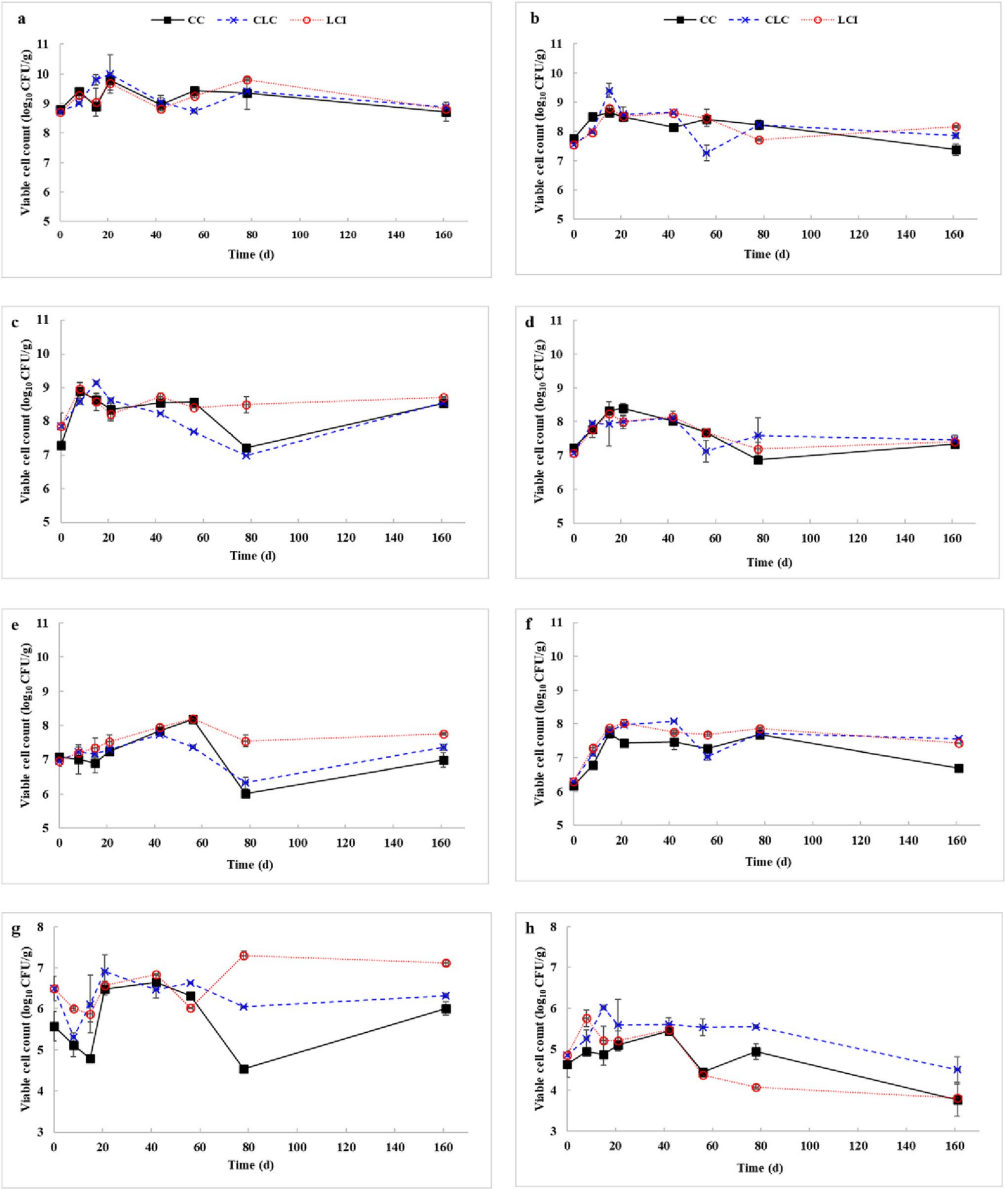
Changes in viable counts of various microbial groups in different types of composts (*CC* control compost, *CLC* control lavender waste compost, *LCI* lavender waste compost with bacterial inoculation; **a** mesophilic microorganisms, **b** thermophilic microorganisms, **c** mesophilic cellulolytic bacteria, **d** thermophilic cellulolytic bacteria, **e** mesophilic streptomycetes, **f** thermophilic streptomycetes, **g** mesophilic fungi, **h** thermophilic fungi); all data points are means ± SD based on three observations

Large and diverse communities of bacteria, actinomycetes, and fungi are actively involved in the conversion and decomposition of lignocellulosic materials (Atif et al. 2020; Varma et al. 2017). The changes in microbial viability and activity throughout the biodegradation of composting mixtures are illustrated in Fig. 2. The results show that bacteria were dominant during the whole composting process. The viable counts of mesophilic microorganisms only varied slightly despite the relatively high temperatures. The highest numbers were observed on day 21 in all three types of composts (Fig. 2a). The various treatments had different effects on the levels of thermophilic microbes (Fig. 2b). The initial viable count of thermophiles was higher in CC than in CLC or LCI. However, due to the high core temperature, their density greatly increased in CLC over the first 15 days. Thermophilic microorganisms were present at concentrations of 2.6 × 10^7^ CFU/g, 7.4 × 10^7^ CFU/g, and 1.5 × 10^8^ CFU/g in CC, CLC, and LCI, respectively, at the end of composting. The high levels of post-extraction lavender waste in CLC and LCI positively influenced the overall microbial activity.

Cellulolytic bacteria were present at high numbers (i.e., 10^7^–10^8^ CFU/g) in the composting materials on day 0. The maximum viable counts of mesophilic cellulose degraders were recorded on day 8 in CC and LCI and a week later in CLC (Fig. 2c). Inoculation with the mixed culture of *C. flavigena* NCAIM B.01383 and *S. viridosporus* NCAIM B.02369 was shown to result in elevated mesophilic cellulolytic bacteria counts in LCI during the cooling phase (between days 56 and 161), thus providing improved conditions for compost maturation. From day 42 until the end of composting, the levels of mesophilic cellulose-degrading bacteria were lower (*p* < 0.05) in CLC than in LCI (Fig. 2c). In contrast, there were no considerable differences in the concentrations of thermophilic cellulose degraders among the three treatments throughout the entire duration of composting (Fig. 2d).

As shown in Fig. 2e, the viable counts of mesophilic streptomycetes increased relatively slowly at first, and then reached peak level on day 42 (in CLC) or day 56 (in CC and LCI). The highest value of 1.6 × 10^8^ CFU/g was observed in LCI on day 56. Inoculation with spore-forming and lignocellulose-degrading *S. viridosporus* NCAIM B.02369 resulted in significantly elevated levels (*p* < 0.05) of mesophilic streptomycetes in LCI, compared to the other two types of composts, during the cooling and maturation phases. As far as thermophilic streptomycetes are concerned, their lowest viable numbers were found in CC at almost all sampling times (Fig. 2f). Overall, the use of lavender waste and bacterial inoculation increased the viability and activity of streptomycetes during the composting process.

In the early thermophilic stage, fungal population sizes were basically determined by temperature. The presence of lavender waste in the composting material positively affected the initial viability of mesophilic fungi (Fig. 2g). Because of increasing compost temperatures, mesophilic fungal counts declined temporarily during the first 8 days in CLC and 15 days in CC and LCI. In contrast, the activity of thermophilic fungi was enhanced over the same period (Fig. 2h). The beneficial effects of lavender waste addition and bacterial inoculation on mesophilic fungal viability were particularly visible during the cooling phase. Thermophilic fungal counts showed a general downward trend in CLC and LCI from day 56 until the completion of composting. However, it is noteworthy that the viable numbers of thermophilic fungi were significantly higher in CLC than in LCI over the final 105 days. Core temperature declined in all three types of composts after day 78 (Fig. 1), and this resulted in decreasing the viable counts of thermophilic fungi while increasing, or at least maintaining, the viability of mesophiles. Overall, the results of microbiological analyses demonstrated that the mixed culture of *C. flavigena* and *S. viridosporus* could regulate the indigenous microbiota, thereby promoting biodegradation.

Composts were also tested for potential human pathogens, such as *Salmonella* spp. and *E. coli* (data not shown). At the start of composting, all treatments were positive for both pathogens. With the progress of time, *E. coli* counts declined gradually in all three types of composts. LCI was free from *E. coli* by day 56, whereas CC and CLC by day 78. As from day 42, no salmonellae were detected in any compost samples. All in all, post-extraction lavender waste failed to show specific antibacterial activity against the potentially pathogenic enterobacteria tested in this study.

### Germination index

**Fig. 3:**
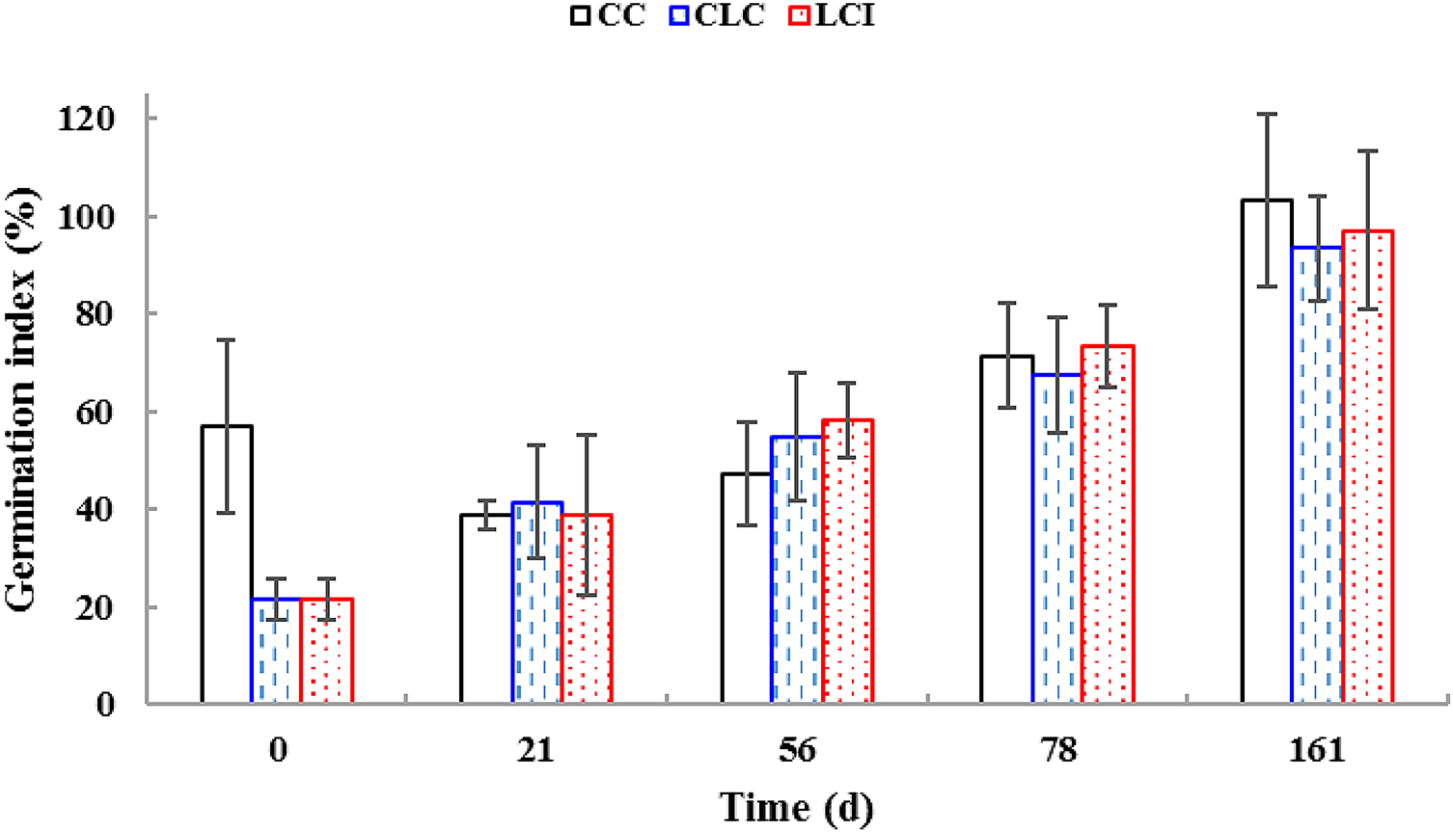
Changes in germination index of *Brassica rapa* subsp. *chinensis* seeds during the composting process (*CC* control compost, *CLC* control lavender waste compost, *LCI* lavender waste compost with bacterial inoculation); values are means ± SD based on three observations

GI is one of the most important parameters used to characterize and evaluate compost maturity. The initial GI values for CC, CLC, and LCI were 56.9, 21.4, and 21.4%, respectively (Fig. 3). From day 21 to day 78, the GI values of all composts increased gradually (67.5–73.8%), indicating that the composts had an acceptable degree of maturity (> 50%), but they still contained phytotoxic compounds influencing seed germination and root growth (Jagadabhi et al. 2019; Jiménez and Garcia 1989).

Final GI values ranged from 93.4 to 103% (Fig. 3), demonstrating that the composts were mature and free from phytotoxins. CC was especially capable of stimulating the germination of *B. rapa* subsp. *chinensis* seeds. LCI was superior to CLC in terms of GI, which must have been due to its increased TN and decreased OM contents (Table 2). Bacterial inoculation only slightly improved the maturity of the finished herbal compost.

### Compost maturity

Composting is the conversion of OM to a mature and biologically stable substance (Cooperband 2000). Mature compost can be safely and easily transported, stored, and applied to the field without harming the environment (van Heerden et al. 2002). In this research, temperature, C/N ratio, GI, and the presence or absence of potentially pathogenic enterobacteria (i.e., *E. coli* and *Salmonella* spp.) were chosen as major maturity indicators. In other studies, temperature was monitored and used to evaluate compost maturity because an extended thermophilic stage usually results in a high-quality and safe final product (Gao et al. 2015). However, our results showed that an extension of the thermophilic phase could improve neither C/N ratio nor GI. Only the lavender waste-containing composts had core temperatures exceeding 55 °C for more than three consecutive days, which is the minimum requirement for effective disinfection (Zeng et al. 2010).

C/N ratio is widely used as an essential indicator of compost maturity (Voběrková et al. 2017). At the end of composting, LCI had a decreased value (*p* < 0.05) compared to the other two products, but the final C/N ratio of all three treatments ranged approximately from 17 to 19. C/N ratios below 20 are typical of properly matured compost (Gou et al. 2017; van Heerden et al. 2002). According to Zucconi et al. (1981), GI values exceeding 80% are indicative of the disappearance of phytotoxic compounds from compost. All composts were free from phytotoxins on day 161. CC had the highest final GI of the three types of composts. Although lavender waste addition negatively influenced this maturity indicator, bacterial inoculation was capable of mitigating the adverse effects of inhibitory residues. In addition to physical and chemical characteristics, a variety of biological properties are also considered in assessing the utility value of compost. Good biological properties include the absence of potential human pathogens in the final composting product (Bohacz 2019b). At completion of composting, all three types of product were free from salmonellae and *E. coli* even though the temperature requirements were not met in CC.

## Conclusions

The use of post-extraction lavender waste positively influenced the 161-day-long composting process by extending the thermophilic stage, elevating the viable counts of beneficial bacteria and fungi, and accelerating the degradation of OM. However, negative effects were also observed, such as an increased C/N ratio and a decreased GI. Inoculation with the mixed culture of *C. flavigena* NCAIM B.01383 and *S. viridosporus* NCAIM B.02369 improved the efficiency of biodegradation and preserved the nitrogen content. The bacterial inoculant reduced the C/N ratio and enhanced the GI of the herbal compost. The *Salmonella*- and *E. coli*-free final composting products were mature and stable. These findings have demonstrated that lavender waste is a raw material suitable for composting, even though it tends to negatively influence the quality of the final product. The adverse effects of herbal residues can be reduced substantially by inoculation with specific bacteria.
